# Antibiotic therapy for bacterial pneumonia

**DOI:** 10.1186/s40780-024-00367-5

**Published:** 2024-07-30

**Authors:** Hideo Kato

**Affiliations:** 1 Department of Pharmacy, Mie University Hospital, Mie, 514-8507 Japan; 2 Department of Clinical Pharmaceutics, Division of Clinical Medical Science, Mie University Graduate School of Medicine, Mie, Japan

**Keywords:** Bacterial pneumonia, Antibiotic therapy, Ceftriaxone, Secondary bacterial pneumonia

## Abstract

Pneumonia is a common infection in patients of all ages. Determining its etiology and selecting antibiotic therapy are challenging for physicians in both private practice and hospitals. Moreover, the coronavirus disease pandemic revealed the importance of prevention and treatment of secondary bacterial pneumonia in patients hospitalized with viral respiratory infections. This review focuses on the types of bacteria that cause pneumonia and provides new insights into antibiotic therapy for bacterial pneumonia. Moreover, it also reviews the current state of knowledge regarding secondary bacterial pneumonia.

## Background

Pneumonia is a disease associated with a high morbidity and mortality rate worldwide, and the incidence is increasing, particularly in immunocompromised individuals, children, and older adults [1]. Pneumonia is defined as the presence of new lung infiltrates with clinical evidence that the infiltrate is of infectious origin, such as new onset of fever, purulent sputum, leukocytosis, and decline in oxygenation [2]. The American Thoracic Society (ATS) and Infectious Diseases Society of America (IDSA) categorize pneumonia as community-acquired pneumonia (CAP) and hospital-acquired pneumonia (HAP), based on the timing of the acquisition [3].

CAP is an acute infection of the pulmonary parenchyma that is acquired outside healthcare facilities. HAP is not present at the time of hospital admission, but has an onset 48 h or more after hospital admission. A substantial proportion of cases of HAP is caused by antibiotic-resistant bacteria, and the prevalence of antibiotic resistance among cases of CAP is also increasing. The incidence of CAP among adults is 14 cases per 1,000 per year [4], and over 50% of cases require hospitalization [5]. The World Health Organization reported that CAP accounts for 4 million deaths per year and 7% of the total annual mortality rate [6]. The economic burden of the CAP is also high, costing an estimated EUR 10.1 billion per year [7]. Similarly, HAP is the main cause of death from nosocomial infection, with an incidence of 5 to 10 cases per 1,000 hospital admissions [8] and an estimated mortality rate of 20–30% [9]. Therefore, bacterial pneumonia warrants attention because it not only threatens the health of individuals but also increases the burden on the national economy.

The microbial etiology of pneumonia includes both bacteria and viruses. During the 1918 H1N1 influenza A virus pandemic, over half the individuals with influenza developed secondary bacterial pneumonia [10]. Even though antibiotics have subsequently been developed, the incidence of secondary bacterial pneumonia remains high in patients with viral pneumonia, and results in increased disease severity. Viral pneumonia complicated by secondary bacterial pneumonia is associated with higher morbidity and mortality compared with viral pneumonia alone in older adults and patients with chronic illnesses [11].

Notwithstanding the considerable global impact, bacterial pneumonia is a particularly important health problem in Japan because its aging population. This review provides details on the types of bacteria that cause pneumonia and provides new insights into antibiotic therapy for bacterial pneumonia. Moreover, the current state of knowledge regarding secondary bacterial pneumonia is also reviewed.

## Bacteria that cause pneumonia

Timely and accurate identification of the pathogens causing pneumonia is critical for the initiation of antibiotic therapy; however, identifying the causative pathogen is challenging in clinical settings [12]. The “gold standard” for determining the etiology is the detection of respiratory pathogens in specimens obtained directly from the lungs by bronchoalveolar lavage, pleural fluid sampling, lung biopsy, or aspiration [13]. Sputum and tracheal aspirates obtained from the lower respiratory tract have a high probability of contamination from by upper respiratory tract bacteria; therefore, pathogens from specimens distant from the site of infection, such as blood and urine, are preferred to sputum and tracheal aspirates. However, the test results from these specimens must be carefully interpreted because no diagnostic method applied to non-pulmonary specimens has both high sensitivity and specificity for identifying the pathogen [12].

### Causative pathogens by country worldwide

There are a wide variety of pneumonia-related pathogens [14]. The most common causes of bacterial pneumonia are *Streptococcus pneumoniae*, *Haemophilus influenzae*, *Klebsiella pneumoniae*, *Pseudomonas aeruginosa*, *Escherichia coli*, and *Staphylococcus aureus* [15]. In Spain, *S. pneumoniae* is the leading cause of bacterial pneumonia, accounting for 31.7% of all cases [16]. In the United Kingdom, *S. pneumoniae* is the most common species associated with pneumonia (30%), followed by *H. influenzae* (19%) [17].

Shoar et al. [18] conducted a systematic review of confirmed cases of pneumonia in adults in developed countries. The review included 146 articles that reported on a total 82,674 patients with pneumonia. *S. pneumoniae* was the most common cause of pneumonia during the entire study period, and was identified, in 33–50% of all cases regardless of the microbiological technique used for identification. *H. influenzae* was the second most common cause (7–16% of cases). In contrast to developed countries, the most common pathogen identified in a study of bacterial pneumonia in Ethiopia was *K. pneumoniae*, with an overall prevalence of 22.0%, followed by *S. pneumoniae* (17.0%), *S. aureus* (14.5%), *P. aeruginosa* (10.0%), and *E. coli* (9.8%) [19]. *K. pneumoniae* has also been reported to be the leading cause of bacterial pneumonia in Cambodia (26.9%), Nepal (27.0%), and Nigeria (23.0%) [20–22]. Because of the differences among countries, routine screening to identify the pathogen is necessary for management of patients with suspected pneumonia to select appropriate antibiotic therapy and to prevent the emergence of antimicrobial resistance.

### Causative pathogens in Japan

In Japan, surveillance of causative respiratory pathogens has been continuously conducted during three periods (1994–1997; 2001–2004; and 2008–2010). *S. pneumoniae* was the most common pathogen identified in all three periods (1994 − 1997, 23.0%; 2001–2004, 23.8%; 2008–2010, 25.9%), with a prevalence similar to that reported in Western countries [23–25]. Recently, Fujikura et al. [26] conducted a systematic review on the epidemiology of pneumonia in Japan in which they reviewed all published studies and epidemiological surveys on the isolation of pathogens in patients with pneumonia in Japan. The authors identified 56 eligible articles reporting on a total of 17,095 cases of bacterial pneumonia. Similar to previous studies [23–25], *S. pneumoniae* was the most common pathogen (20.0%), followed by *H. influenzae* (10.8%). In patients with pneumonia requiring hospitalization, *S. pneumoniae* was also the most frequently isolated pathogen (16.2%), followed by *H. influenzae* (6.9%). Moreover, the prevalence of *S. pneumoniae* was similar in studies published before 2000 and those published after 2010.

### Antibiotic-resistant *S. pneumoniae*

Antibiotic resistance is a major concern in selecting treatment for bacterial pneumonia [27]. Antibiotic resistance has become a problem due to the emergence and dissemination of antibiotic-resistant pathogens in hospitals and community settings, inappropriate antibiotic use, and the overconsumption of antibiotics [28]. In China and Ethiopia, most *S. pneumoniae* isolates are highly resistant to erythromycin, azithromycin, and clindamycin [29, 30]. In Japan, the Japanese Society of Chemotherapy, Japanese Association for Infectious Diseases, and Japanese Society for Clinical Microbiology have conducted nationwide surveillance of the antimicrobial susceptibility of bacterial respiratory pathogens isolated from patients with lower respiratory infections [31]. Forty-two medical institutions participated in the surveillance and 264 *S pneumoniae* strains were evaluated. All strains were susceptible to benzylpenicillin, with a minimum inhibitory concentration (MIC) of less than 2 mg/L. Among β-lactam antibiotics, the 90% MIC (MIC_90_) results of cephems other than cefaclor, cefmetazole, and ceftazidime were 0.5–2 mg/L and those of carbapenems were less than 0.25 mg/L. The MIC_90_ results of fluoroquinolones were 0.06–4 mg/L, and the MICs of garenoxacin and sitafloxacin against all strains were less than 1 mg/L. In contrast, strains resistant to macrolides and clindamycin were common, with MIC_90_ results of over 128 mg/L. The pattern of antibiotic resistance in *S. pneumoniae* is attributable to empirical antibiotic administration and poor adherence to treatment guidelines. Therefore, specific antibiotic therapy is fundamental to the management and control of bacterial pneumonia.

## Antibiotic therapy

Antibiotic therapy for patients with suspected bacterial pneumonia should be appropriate and should be administered as early as possible. Current guidelines recommend the use of β-lactam and β-lactamase inhibitor combinations, especially sulbactam-ampicillin (SAM) and ceftriaxone, for the initial treatment of pneumonia [2, 32, 33]. However, because the diagnosis of pneumonia is uncertain in some cases, patients with suspected pneumonia are prescribed empiric antibiotic therapy prior to identification of a bacterial pathogen to prevent rapid clinical deterioration and the need for hospital admission.

### Ceftriaxone vs. broad-spectrum antibiotics

Some prognostic guidelines recommend the use of piperacillin-tazobactam (TZP) and carbapenems for the treatment of hospitalized patients with pneumonia [34, 35]. TZP and carbapenems are needed in the treatment of severe or very severe pneumonia. However, a previous study has shown that broad-spectrum antibiotics such as TZP and carbapenems are used as an empiric treatment in 50–68% of patients hospitalized with pneumonia in Japan [36]. The use of these broad-spectrum antibiotics is associated with several problems. First, because broad-spectrum antibiotics have anti-anaerobic activity, the use of broad-spectrum antibiotic therapy could cause the emergence of *Clostridioides difficile* infections [37, 38]. The use of broad-spectrum antibiotics leads to an increase in antimicrobial resistance rates and medical costs. Thus, ceftriaxone, which could cover almost every pathogen causing pneumonia, is considered to be one of the most useful antibiotics in the treatment of pneumonia. A single-center retrospective study was conducted in patients with pneumonia to evaluate whether ceftriaxone is as effective as broad-spectrum antibiotics (TZP and carbapenems) for the treatment of pneumonia [39]. The 30-day mortality rate was 0% in both groups. There were no differences in the incidence of initial treatment failure (ceftriaxone vs. broad-spectrum antibiotics, 4.3% vs. 0%, *p* = 0.312), inappropriate treatment (23.1% vs. 26.7%, *p* = 0.827), mean duration of antibiotic therapy (12.3 ± 7.4 days vs. 12.5 ± 5.4 days, *p* = 0.928), or mean length of hospital stay (17.0 ± 13.4 days vs. 17.0 ± 7.7 days, *p* = 0.980) in the two groups. However, the medical costs were considerably higher in the broad-spectrum antibiotic group than in the ceftriaxone group (8,678 Japanese yen vs. 35,582 Japanese yen, *p* < 0.001). These findings demonstrate that ceftriaxone is an effective treatment for pneumonia and is not inferior to broad-spectrum antibiotics.

### Ceftriaxone vs. SAM

Ceftriaxone is the most commonly used antibiotic for the treatment of pneumonia because it requires less frequent administration than that of alternative antibiotics, does not require a dose adjustment in patients with mild and moderate impaired renal function, and is suitable for use as an alternative antibiotic in patients who are allergic to penicillin [40, 41]. Ceftriaxone has a spectrum of activity similar to that of SAM against the predominant bacterial pathogens that cause pneumonia [42] However, ceftriaxone does not target the full spectrum of oral anaerobes that cause pneumonia [34, 43]. To date, only one meta-analysis has provided comprehensive evidence of the effectiveness of antibiotics in the treatment of pneumonia [6]. However, the meta-analysis did not compare the effectiveness of ceftriaxone and SAM for the treatment of pneumonia. Hence, a meta-analysis was subsequently conducted to compare the effectiveness of ceftriaxone and SAM as the initial treatment for pneumonia [44]. Four studies were included in the meta-analysis. The four studies included a total of 390 patients who received ceftriaxone, and 604 patients who received SAM. There were no significant differences in mortality and clinical cure rates between patients receiving ceftriaxone and SAM (ceftriaxone vs. SAM: mortality, 5.6% vs. 11.4%, odds ratio (OR): 1.85, 95% confidence interval (CI): 0.57–5.96; clinical cure rate, 87.5% vs. 91.8%, OR: 1.08, 95% CI: 0.18–6.44). Therefore, the meta-analysis concluded that ceftriaxone was not inferior to SAM as an initial treatment for pneumonia. However, it is necessary to consider the use of SAM in patients who have an unfavorable clinical course with ceftriaxone therapy, because β-lactamase-positive strains have been detected in 80–85% of oral anaerobes associated with pneumonia [45].

### Optimal dosing regimen of ceftriaxone

Ceftriaxone, a third-generation cephalosporin, has a long plasmatic half-life [46], and thus, it can be administered once daily [47]. A 2 g dose administered once daily is frequently used in clinical settings [48, 49]. However, the Sanford Guide for Antimicrobial Therapy [50] suggests administering a 1 g dose of ceftriaxone twice daily. Recently, a single-center retrospective study and a basic study compared the effectiveness and safety of ceftriaxone administered as either a 1 g dose twice daily or a 2 g dose once daily, for the treatment of pneumonia [51, 52].

In a retrospective study [51], 61 patients were included (1 g twice daily, *n* = 33; 2 g once daily, *n* = 28). Only five patients receiving 1 g twice daily failed treatment for pneumonia, with a significant difference between the two dosing regimens (1 g administered twice daily vs. 2 g administered once daily, 15.2% vs. 0%, *p* = 0.032). The percentages of patients who were afebrile (defined as a body temperature [BT] < 37.0 °C) and had C-reactive protein (CRP) levels < 60% of the baseline at the end of therapy were higher in patients who received 2 g once daily than in those who received 1 g twice daily (afebrile, 45.5% vs. 78.3%, *p* = 0.014; decreased CRP level, 25.0% vs. 60.7%, *p* = 0.005). Moreover, changes in white blood cell (WBC) count, BT, and CRP levels were investigated for 2 weeks after the initiation of ceftriaxone therapy. Patients who received 2 g once daily showed significant decreases in WBC, BT, and CRP from 4 to 7 day after the initiation of ceftriaxone therapy, while patients who received 1 g twice daily showed significant decreases in BT and CRP from 4 to 7 days and 8–14 days, respectively. And CRP level and BT on 4–7 day in patients who received 2 g once daily were significantly lower than those in patients who received 1 g twice daily (2 g once daily vs. 1 g twice daily: CRP, 7.7 ± 6.2 mg/dL vs. 5.3 ± 4.1 mg/dL, *p* = 0.037; body temperature, 37.3 ± 0.7 °C vs. 37.1 ± 0.6 °C, *p* = 0.052). In contrast, the percentage of patients with abnormal liver function test results did not differ significantly between the two dosing regimens. However, the percentage of patients diagnosed with choleliths during ceftriaxone therapy was higher in those who received 2 g once daily than in those who received 1 g twice daily (31.3% vs. 9.1%; *p* = 0.174).

In a basic science study [52], the antimicrobial activities of two dosing regimens against *S. pneumoniae* with MICs of 1, 2, and 4 mg/L, were assessed using a murine model of pneumonia. The 2 g once daily regimen showed significantly higher antimicrobial activity against *S. pneumoniae* with MICs of 1 and 2 mg/L compared with the 1 g twice daily regimen (1 mg/L, − 5.14 ± 0.19 Δlog_10_ colony-forming unit (cfu)/lungs vs. −3.47 ± 0.17 Δlog_10_ cfu/lungs, *p* < 0.001; 2 mg/L, − 3.41 ± 0.31 Δ log_10_ cfu/lungs vs. −2.71 ± 0.37 Δlog_10_ cfu/lungs, *p* = 0.027). In contrast, no significant difference in antimicrobial activity was observed against *S. pneumoniae* with a MIC of 4 mg/L between the two dosing regimens (− 0.33 ± 0.18 Δlog_10_ cfu/lungs versus − 0.42 ± 0.37 Δlog_10_ cfu/lungs, *p* = 0.684).

These studies demonstrated that a 2 g once daily regimen of ceftriaxone showed an improvement of clinical responses and early reduction of inflammatory markers and that the 2 g once daily regimen was effective in a murine model of pneumonia caused by *S. pneumoniae* with a MIC of ≤ 2 mg/L. Therefore, the 2 g once daily regimen was concluded to be a favorable dosing regimen for the initial treatment of pneumonia.

## Secondary bacterial pneumonia following viral pneumonia

### Causative bacterial pathogens

It was recently reported that the proliferation of viruses in lung tissues creates a suitable environment for bacterial pathogen colonization [53]. The onset of secondary bacterial pneumonia may cause a worsening of the patient’s clinical condition [54]. To date, *S. pneumoniae* has been the predominant bacterial pathogen associated with influenza virus pandemics, accounting for one-quarter to one-half of severe and fatal cases [11, 55–57]. In patients with coronavirus disease 2019 (COVID-19), *S. pneumoniae* is also the most common cause of secondary bacterial pneumonia [54]. *S. pneumoniae* is a common part of the nasopharyngeal flora, with a prevalence of colonization of 40–95% in infants and 10–25% in adults [58]. Therefore, antibiotic therapies targeting *S. pneumoniae* should be considered in patients with secondary bacterial pneumonia.

### Strategy of antibiotic therapy

The likelihood of combined viral and bacterial pneumonia is low in patients with mild-to-moderate viral pneumonia, and antibiotics can be safely withheld [59]. In the absence of supporting evidence of bacterial pneumonia, antibiotics should not be initiated even in patients with progressive respiratory distress [59]. However, data regarding the role of secondary bacterial pneumonia in acute respiratory decompensation are limited. Therefore, guideline-driven empirical antibiotic use may be reasonable until secondary bacterial pneumonia has been ruled out [59]. Of patients hospitalized with viral pneumonia, 56.6% have been reported to be treated with early empirical antibiotics [60]. In our hospital, ceftriaxone is recommended as an initial antibiotic therapy in patients hospitalized with COVID-19. A study has shown that ceftriaxone therapy is effective and safe in patients hospitalized with COVID-19 (under review). However, current studies provide insufficient evidence to support the routine use of antibiotics in patients hospitalized with COVID-19 [61]. Therefore, further research is needed to establish the most appropriate antibiotic therapy for the prevention and treatment of secondary bacterial pneumonia in patients with COVID-19.

### Prevention of bacterial pneumonia

Prevention may be the best approach to the management of secondary bacterial pneumonia. Vaccination is the preferred means to prevent pneumonia. Seasonal vaccination with inactivated influenza vaccine has been shown to decrease the incidence of hospitalization and death due to pneumonia [62], and the use of a conjugate pneumococcal vaccine has been shown to prevent viral lower respiratory tract infections [62]. However, the development of mutations and new subtypes in has weakened the effectiveness of vaccination of *S. pneumoniae*, influenza, and COVID-19. Therefore, the development of new methods to prevent the onset of pneumonia and alleviate its severity are urgently needed.

## Conclusions

Because bacterial and viral pneumonia have high mortality rates, knowledge of possible pathogens and their therapeutic implications is essential for providing adequate antibiotic therapy. In developed countries, including Japan, *S. pneumoniae* is the leading cause of both primary and secondary bacterial pneumonia; therefore, broad-spectrum antibiotics effective against *S. pneumoniae* need to be considered in the initial treatment of bacterial pneumonia. An optimal dosing regimen for the initial treatment of pneumonia with ceftriaxone, which is one of the antibiotics recommended in the guidelines, has been established, and ceftriaxone has been shown to be effective and safe. However, further research is required to establish a new, better approaches for preventing secondary bacterial pneumonia.

## Data Availability

Not applicable.

## Abbreviations

ATS: American Thoracic Society
BT: body temperature
CAP: community-acquired pneumonia
cfu: colony-forming unit
CI: confidence interval
COVID-19: coronavirus disease 2019
CRP: C-reactive protein
HAP: hospital-acquired pneumonia
IDSA: Infectious Diseases Society of America
MIC: minimum inhibitory concentration
OR: odds ratio
SAM: sulbactam-ampicillin
TZP: piperacillin-tazobactam
WBC: white blood cell
